# Optimizing the Therapeutic Index of sdAb-Based Radiopharmaceuticals Using Pretargeting

**DOI:** 10.2967/jnumed.124.267624

**Published:** 2024-10

**Authors:** Sophie Poty, Laura Ordas, Yana Dekempeneer, Ali Asghar Parach, Laurent Navarro, Francis Santens, Nina Dumauthioz, Manuel Bardiès, Tony Lahoutte, Matthias D’Huyvetter, Jean-Pierre Pouget

**Affiliations:** 1 Institut de Recherche en Cancérologie de Montpellier, Université de Montpellier, INSERM, U1194, Équipe labellisée Ligue contre le cancer, Montpellier, France; and; 2 Precirix NV/SA, Brussels, Belgium

**Keywords:** pretargeting, single-domain antibody, FAP

## Abstract

Single-domain antibodies (sdAbs) demonstrate favorable pharmacokinetic profiles for molecular imaging applications. However, their renal excretion and retention are obstacles for applications in targeted radionuclide therapy (TRT). **Methods:** Using a click-chemistry–based pretargeting approach, we aimed to reduce kidney retention of a fibroblast activation protein α (FAP)–targeted sdAb, 4AH29, for ^177^Lu-TRT. Key pretargeting parameters (sdAb-injected mass and lag time) were optimized in healthy mice and U87MG (FAP^+^) xenografts. A TRT study in a pancreatic ductal adenocarcinoma (PDAC) patient-derived xenograft (PDX) model was performed as a pilot study for sdAb-based pretargeting applications. **Results:** Modification of 4AH29 with *trans*-cyclooctene (TCO) moieties did not modify the sdAb pharmacokinetic profile. A 200-µg injected mass of 4AH29-TCO and an 8-h lag time for the injection of [^177^Lu]Lu-DOTA-PEG_7_-tetrazine resulted in the highest kidney therapeutic index (2.0 ± 0.4), which was 5-fold higher than that of [^177^Lu]Lu-DOTA-4AH29 (0.4 ± 0.1). FAP expression in the tumor microenvironment was validated in a PDAC PDX model with both immunohistochemistry and PET/CT imaging. Mice treated with the pretargeting high-activity approach (4AH29-TCO + [^177^Lu]Lu-DOTA-PEG_7_-tetrazine; 3 × 88 MBq, 1 injection per week for 3 wk) demonstrated prolonged survival compared with the vehicle control and conventionally treated ([^177^Lu]Lu-DOTA-4AH29; 3 × 37 MBq, 1 injection per week for 3 wk) mice. Mesangial expansion was reported in 7 of 10 mice in the conventional cohort, suggesting treatment-related kidney morphologic changes, but was not observed in the pretargeting cohort. **Conclusion:** This study validates pretargeting to mitigate sdAbs’ kidney retention with no observation of morphologic changes on therapy regimen at early time points. Clinical translation of click-chemistry–based pre-TRT is warranted on the basis of its ability to alleviate toxicities related to biovectors’ intrinsic pharmacokinetic profiles. The absence of representative animal models with extensive stroma and high FAP expression on cancer-associated fibroblasts led to a low mean tumor-absorbed dose even with high injected activity and consequently to modest survival benefit in this PDAC PDX.

## Visual Abstract

**Figure:**
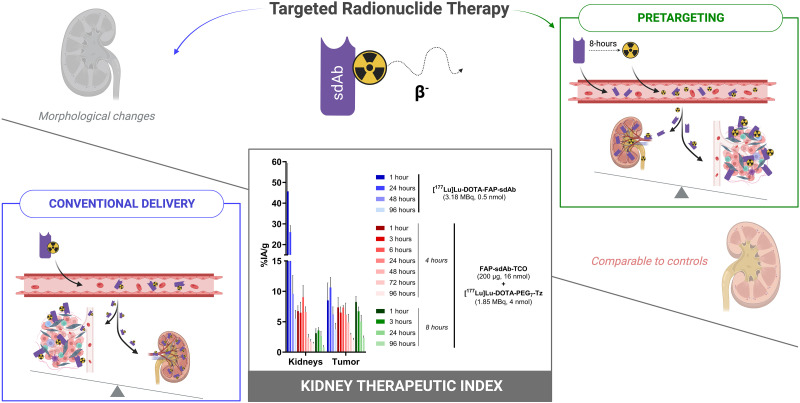

Camelid single-domain antibody (sdAb) fragments (also referred as variable heavy domain of heavy chains or Nanobodies [Ablynx]) show promising features for radiotheranostics development (1). Because of their small size (10–15 kDa), sdAbs demonstrate rapid blood and renal clearance and enhanced tumor penetration. sdAbs are easily produced, and their structure can be modified to introduce amino acids or functional groups for imaging or therapeutic purposes. sdAbs targeting cell-membrane biomarkers were evaluated in preclinical and clinical studies as theranostic agents with a range of radionuclides for PET, SPECT, or therapy (1). A phase I trial assessing a ^68^Ga-sdAb conjugate targeting HER2 in 20 breast cancer patients highlighted the favorable biodistribution profile, safety, and tolerability of sdAb radioconjugates for diagnostic purposes, leading to a phase II trial in breast cancer patients with brain metastases (NCT03331601) (2). Although valuable in the imaging context, sdAb renal clearance is a limitation for application in targeted radionuclide therapy (TRT) because of the potential associated nephrotoxicity. Approaches to reduce sdAb kidney retention were evaluated. Coadministration of gelofusine or lysine resulted in a 40%–50% decrease in kidney retention without affecting tumor uptake (3). sdAb kidney retention is mediated by polar residues in their C-terminal tag; therefore, untagged sdAbs achieved a 70% decrease in kidney retention (4). Finally, a shift from sdAb–radiometal conjugates to radiohalogens led to the fastest renal excretion reported yet, surpassing gelofusine coadministration (5). This preclinical observation was confirmed in a phase I trial with [^131^I]I-GMIB-anti-HER2-sdAb in 6 healthy volunteers and 3 patients with metastatic breast cancer (6). Despite significant improvement, the reported kidney therapeutic index (TI) of sdAb-based radiopharmaceuticals in rodents remains poor (<1.5), and further investigations are warranted.

Pretargeting is a concept based on the sequential administration of a targeting vector and a radioactive payload. The conjugation of the radionuclide to the vector happens in vivo according to different mechanisms, including click chemistry (7). Pretargeting was successfully applied to antibody-based radiopharmaceuticals, resulting in improved tumor-to-background ratios for SPECT/PET imaging and reduced off-target or healthy-tissue toxicities in the context of TRT (8–10). In particular, pretargeted radioimmunotherapy with ^225^Ac displayed reduced hematotoxicity compared with conventional administration approaches, without affecting therapeutic effectiveness (11). Although the methodology was mostly applied to antibodies, recent years have witnessed a shift to smaller vectors such as diabodies, Affibody (Affibody AB) molecules, or bisphosphonates (12–15).

In this context, we applied and optimized this approach to sdAb TRT with the hypothesis that pretargeting should result in decreased kidney uptake, higher tumor-to-kidney ratios, and overall improved TI. A click-chemistry approach was selected because it affords easy and modular functionalization of sdAbs through covalent chemistry. Inverse electron demand Diels–Alder cycloaddition between *trans*-cyclooctene (TCO) and tetrazine was used because of its selectivity, biorthogonality, and rapidity. We optimized sdAb pretargeting using a fibroblast activation protein α (FAP)–targeting sdAb, 4AH29, that was recently validated in vitro and in vivo by our group (16). FAP is a membrane-bound protein, overexpressed in cancer-associated fibroblasts (CAFs), and found in 90% of epithelial cancers. This target has attracted attention in the nuclear medicine community, and radiolabeled FAP inhibitors (FAPIs) were evaluated. [^68^Ga]Ga-FAPI-04 identified tumor lesions across 28 kinds of cancer (17). However, for applications in TRT, most FAPI radiopharmaceuticals faced fast clearance and insufficient tumor retention. To overcome these hurdles, ongoing clinical investigations favor peptides (FAP2286; Novartis) or inhibitors (PNT6555; Lilly) optimized for prolonged retention. Accordingly, the use of larger vectors, such as sdAbs, could mitigate the challenging clearance of FAPIs. 4AH29 previously demonstrated prolonged tumor retention up to 72 h after injection when radiolabeled with ^131^I or ^225^Ac (16).

In this study, we determined the optimal key parameters for sdAb pretargeting, including lag time and sdAb-injected mass to obtain the highest kidney TI. We report the associated mouse dosimetry and, to our knowledge, first in vivo evaluation in a pancreatic ductal adenocarcinoma (PDAC) patient-derived xenograft (PDX) model.

## MATERIALS AND METHODS

C57BL/6J (6 wk) were used as healthy animals. Athymic nude mice (Crl:NU(NCr)-Foxn1nu, 6 wk) were xenografted with 2.5 × 10^6^ U87MG cells in a 50:50 medium-to-Matrigel ratio on the right flank 3 wk before the biodistribution study to reach approximately 100–150 mm^3^ at the start of the study. Swiss nude mice (Crl:NU(Ico)-Foxn1nu, 4–5 wk) were implanted with an approximately 30-mm^3^ PDAC PDX tumor fragment into the intrascapular region to reach approximately 160–170 mm^3^ at the start of the therapy study.

All results in this article are presented as mean ± SD. Information on chemical structures (Supplemental Fig. 1 [supplemental materials are available at http://jnm.snmjournals.org]), bioconjugation, radiochemistry, in vitro and in vivo assays, and dosimetry are available in the supplemental materials (18–20).

## RESULTS

### TCO Functionalization of sdAb Does Not Affect Affinity or Biodistribution

**FIGURE 1.**
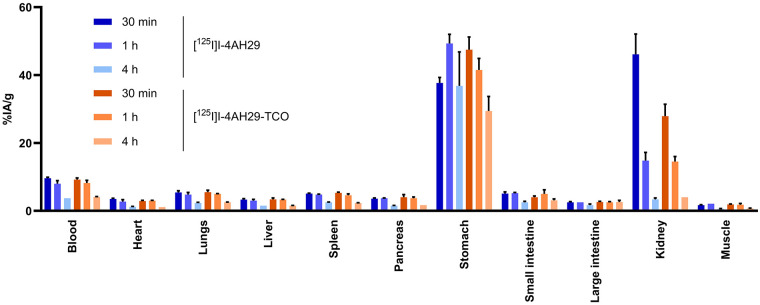
Impact of TCO functionalization on 4AH29’s intrinsic biodistribution with comparison of [^125^I]I-4AH29 and [^125^I]I-4AH29-TCO (10 µg [0.8 nmol], 6 GBq/µmol) in healthy C57BL/6J mice (*n* = 3/cohort).

FAP (4AH29) and control (R3B23) sdAbs were functionalized with TCO moieties (Supplemental Fig. 2). Enzyme-linked immunosorbent assay revealed similar effective concentrations of 50% between 4AH29 (0.55 nM) and 4AH29-TCO (0.58 nM; Supplemental Fig. 3A). The impact of TCO functionalization on 4AH29 antigen binding and its intrinsic biodistribution was further evaluated using radioiodinated sdAbs. Tyrosine residues were radioiodinated randomly to remain close to the sdAb native structure. The antigen binding fraction was determined in cell- or bead-based assays (Supplemental Fig. 3B). Modest antigen binding fractions were observed in cell-based assays because of low (U87MG) and heterogeneous (HEK293–human FAP) FAP expression (Supplemental Fig. 4). Bead-based assays resulted in antigen binding fractions of more than 90%. Similar antigen binding fractions between native and TCO-functionalized sdAbs were reported in all assays, confirming the negligible impact of TCO modification on antigen recognition (21). Biodistribution in healthy mice showed comparable pharmacokinetic profiles between ^125^I radioconjugates with reduced kidney uptake only, 30 min after injection, for [^125^I]I-4AH29-TCO (27.9 ± 3.5 vs. 46.1 ± 6.0 percentage injected activity per gram of tissue [%IA/g]) compared with [^125^I]I-4AH29, which could be due to the reduced net charge of the TCO conjugate (Fig. 1). The stomach uptake is due to released radioiodine and trafficking through tissue expressing Na^+^/I^−^ symporters.

### Optimization of Lag Time and sdAb-TCO–Injected Mass in Healthy Mice

**FIGURE 2.**
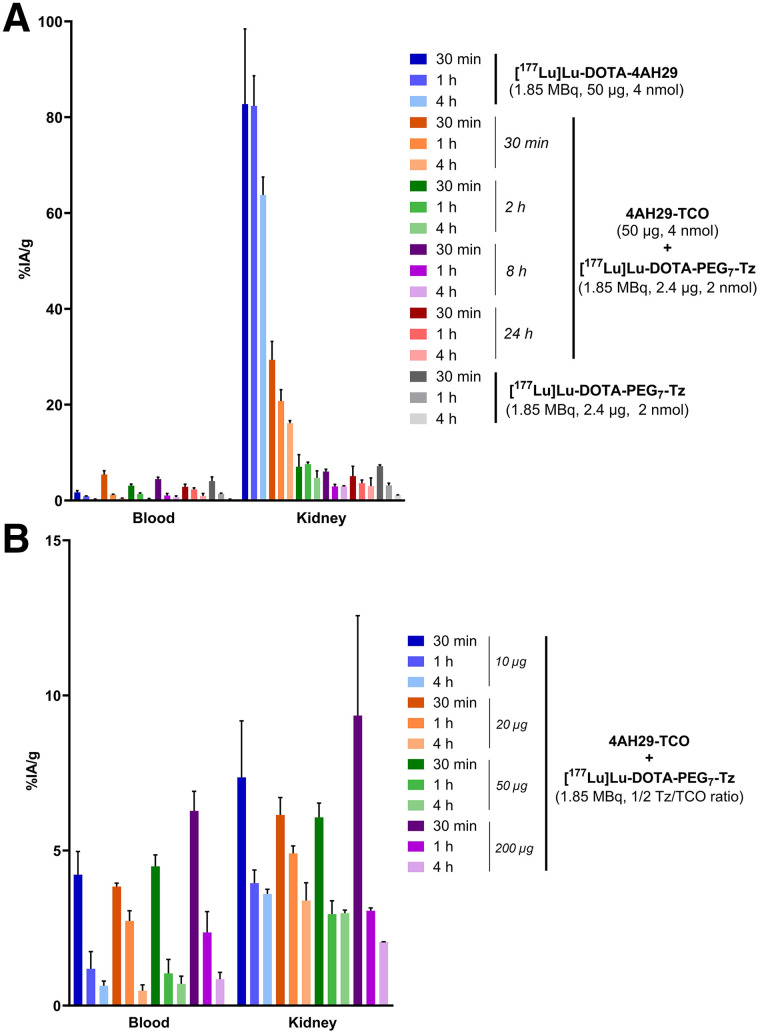
Optimization of pretargeting lag time (A) and injected mass of 4AH29-TCO (B) in healthy C57BL/6J mice (*n* = 4/cohort). Tz = tetrazine.

The application of pretargeting to reduce kidney retention of sdAbs was first validated in healthy mice. Four lag times (30 min, 2 h, 8 h, and 24 h) were evaluated. First, 4AH29-TCO (50 µg, 4 nmol) was injected intravenously. Then, at the set lag time, [^177^Lu]Lu-DOTA-PEG_7_-tetrazine (1.85 MBq, 2 nmol; Supplemental Figs. 5–7) was injected (10*,*^22^). Control groups were administered either [^177^Lu]Lu-DOTA-4AH29 (Supplemental Figs. 8–10) or [^177^Lu]Lu-DOTA-PEG_7_-tetrazine (without preinjection of 4AH29-TCO). Pretargeting resulted in a 3-fold or greater decrease in kidney uptake (Fig. 2A; Supplemental Fig. 11; Supplemental Table 1). The 8-h lag time led to a 23-fold reduction in kidney-cumulated activity compared with [^177^Lu]Lu-DOTA-4AH29 (11.2 ± 0.7 vs. 260.4 ± 11.8 %IA/g·h; Supplemental Fig. 12). We concluded that past a 2-h lag time, 4AH29-TCO is either excreted or metabolized, resulting in a significant decrease in kidney uptake using pretargeting.

Four 4AH29-TCO–injected masses (10–200 µg, 0.8–16 nmol) were assessed with a set 8-h lag time (Fig. 2B; Supplemental Fig. 13; Supplemental Table 2). The highest injected masses, 50 and 200 µg, displayed the lowest kidney-cumulated activity (Supplemental Fig. 14), meaning that a high injected mass was not limiting and that further escalation could be envisioned in tumor-bearing mice.

### Optimization of 4AH29-TCO–Injected Mass in U87MG Subcutaneous Xenografts

**FIGURE 3.**
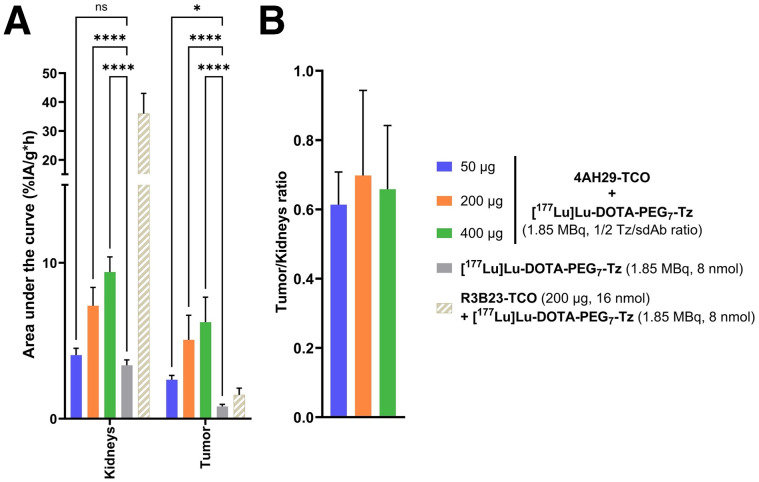
Optimization of 4AH29-TCO–injected mass with 4-h lag time in U87MG subcutaneous xenografts (*n* = 5/cohort). (A) Area under curve for pretargeting (4AH29-TCO + [^177^Lu]Lu-DOTA-PEG_7_-tetrazine) and control groups. Two-way ANOVA analysis with Dunnett multiple comparison test was applied. (B) Tumor-to-kidney area under curve ratio. *Adjusted *P* < 0.05. ****Adjusted *P* < 0.0001. ns = nonsignificant; Tz = tetrazine.

Immunohistochemistry revealed an FAP-positive tumor area of more than 40% in U87MG subcutaneous xenografts (Supplemental Fig. 15). Three 4AH29-TCO–injected masses (50, 200, and 400 µg) were evaluated with a 4-h lag time. In contrast to healthy mice, the largest injected masses displayed the highest and most prolonged kidney uptake and retention (Fig. 3A; Supplemental Fig. 16; Supplemental Table 3). Tumor uptake was also affected by injected masses. The 50-µg cohort exhibited an overall lower tumor area under the curve and faster clearance than those of the 200- and 400-µg cohorts. Compared with control groups, [^177^Lu]Lu-DOTA-PEG_7_-tetrazine injected alone, and pretargeting with irrelevant R3B23-TCO, both 200- and 400-µg cohorts demonstrated a significantly greater area under the curve (Fig. 3A), indicating FAP-specific accumulation. All pretargeting cohorts showed comparable tumor-to-kidney ratios (Fig. 3B). Therefore, an injected mass of 200 µg was chosen to compromise high tumor uptake and low kidney retention.

The impact of the degree of functionalization of 4AH29 with TCO moieties was evaluated. This parameter slightly increased tumor uptake for conjugates with the highest degree of functionalization (2–3 TCOs per sdAb; Supplemental Fig. 17; Supplemental Table 4). The addition of a PEG_4_ spacer between the sdAb and the TCO moiety was attempted with the intention of increasing the accessibility of the TCO moiety. However, it resulted in a 2-fold decrease in tumor uptake compared with the TCO conjugate with no spacer (Supplemental Fig. 18; Supplemental Table 4). Kidney uptake was also reduced, suggesting weakened TCO reactivity. This modification was dismissed.

### Optimization of Pretargeting Lag Time in U87MG Subcutaneous Xenografts

**FIGURE 4.**
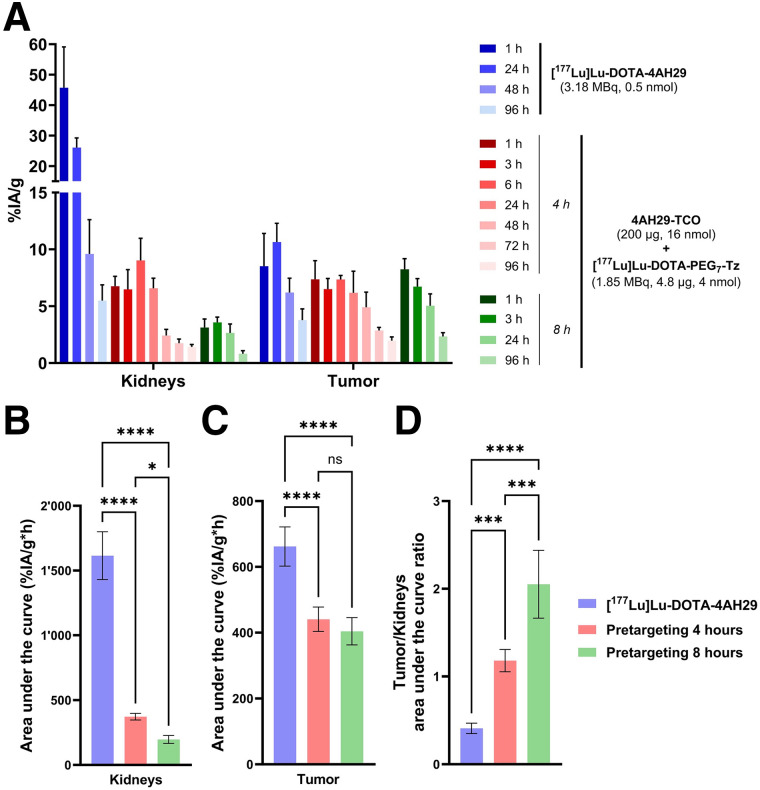
Optimization of pretargeting lag time in U87MG subcutaneous xenografts (*n* = 5/cohort). (A) Kidney and tumor biodistribution profiles for pretargeting with 4- or 8-h lag time and directly radiolabeled sdAb. (B and C) Area under curve for kidneys (B) and tumor (C). (D) Tumor-to-kidney ratio. Ordinary 1-way ANOVA analysis with Šídák correction was applied. *Adjusted *P* < 0.05. ***Adjusted *P* < 0.001. ****Adjusted *P* < 0.0001. ns = nonsignificant; Tz = tetrazine.

The 4- and 8-h lag times were further evaluated in U87MG subcutaneous xenografts. Pretargeting exhibited a remarkable reduction in kidney uptake and area under the curve (Figs. 4A and 4B; Supplemental Fig. 19; Supplemental Table 5). The 8-h lag time resulted in an 8-fold decrease in kidney-cumulated activity compared with [^177^Lu]Lu-DOTA-4AH29 (197 ± 31 vs. 1,615 ± 185 %IA/g·h). Tumor-cumulated activity also showed a decrease with pretargeting, 441 ± 37 and 404 ± 41 %IA/g·h for the 4- and 8-h lag time, compared with 662 ± 60 %IA/g·h for [^177^Lu]Lu-DOTA-4AH29 (Fig. 4C). Despite this latter decrease, pretargeting displayed higher tumor-to-kidney ratios. The 8-h lag resulted in the highest tumor-to-kidney ratio (2.0 ± 0.4), which corresponds to a 5-fold increase compared with [^177^Lu]Lu-DOTA-4AH29 (0.4 ± 0.1). This experiment was repeated, and the pretargeting tumor-to-kidney ratio for the 8-h lag time was confirmed (Supplemental Fig. 20). Coinjection of 4AH29-TCO with gelofusine was attempted to further reduce kidney uptake; however, no improvement of kidney-cumulated activity was observed with this approach (Supplemental Fig. 21).

### Pretargeting Results in a 5-Fold Increase in Kidney TI

Mouse dosimetry was performed using biodistribution data in U87MG xenografts (Table 1; Supplemental Table 6). Pretargeting (200 µg of 4AH29-TCO and 8-h lag time) resulted in an 8.5-fold decrease in the kidneys’ mean absorbed dose (MAD; 15.6 cGy/MBq) compared with [^177^Lu]Lu-DOTA-4AH29 (134.0 cGy/MBq). Kidneys remain the dose-limiting organ for TRT. Pretargeting led to a 1.6-fold decrease in tumor MAD (37.5 vs. 60.0 cGy/MBq). Nevertheless, pretargeting exhibited a 5-fold increase in kidney TI compared with [^177^Lu]Lu-DOTA-4AH29, a major improvement for sdAb TRT.

**TABLE 1.** Pretargeting Mouse Dosimetry Compared with [^177^Lu]Lu-DOTA-4AH29

	4AH29-TCO + [^177^Lu]Lu-DOTA-PEG_7_-tetrazine, 8-h lag time		[^177^Lu]Lu-DOTA-4AH29	
Organ	cGy/MBq	TI	cGy/MBq	TI
Blood	0.55	68	0.54	110
Kidneys	15.64	2.40	134.02	0.45
Tumor	37.54	—	60.03	—

MADs were calculated using biodistribution data reported in Figure 4.

### Evaluation of a PDAC PDX Model for FAP Expression

**FIGURE 5.**
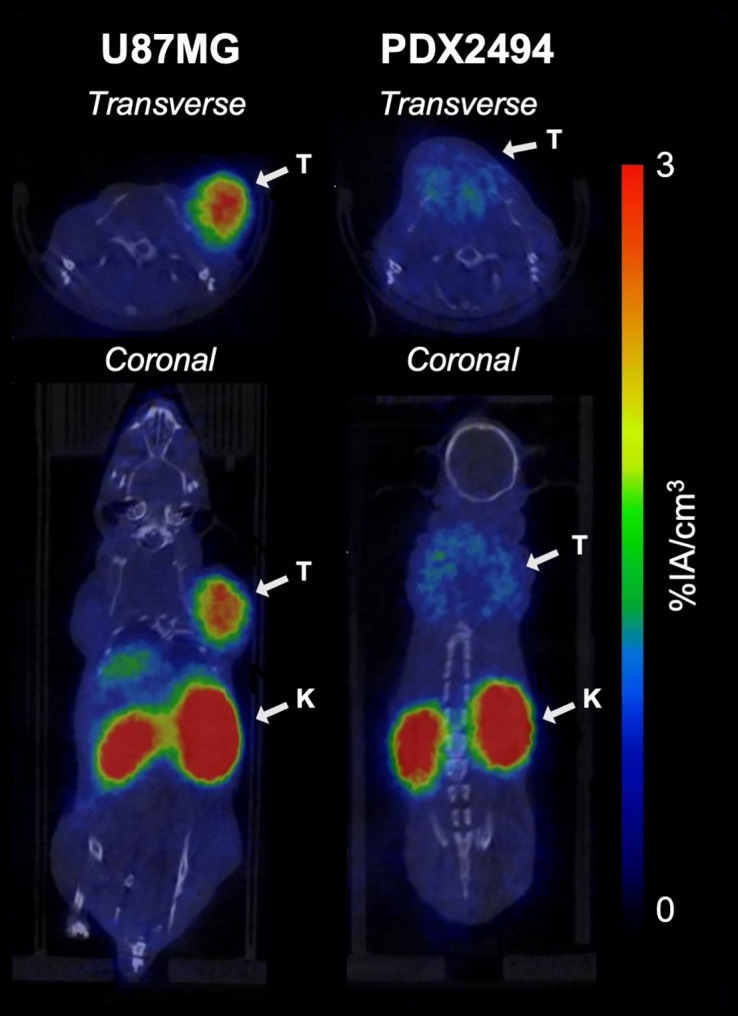
PET/CT imaging of FAP expression in U87MG subcutaneous xenografts (*n* = 4) and PDX2494 intrascapular xenografts (*n* = 10). Representative transverse and coronal PET/CT slices of [^68^Ga]Ga-DOTA-4AH29 (18–22 MBq/mouse and 0.4–0.5 nmol [5.3–6.7 µg]/mouse), 1 h after injection. Arrows indicate tumors (T) and kidneys (K).

Because FAP is mostly expressed on CAFs, we aimed to identify a relevant model to validate the therapeutic potential of our pretargeting approach. PDXs available at the Institut de Recherche en Cancérologie de Montpellier were screened by immunohistochemistry. A PDAC PDX, PDX2494, revealed positive staining for both α-smooth muscle actin and FAP in the tumor microenvironment, with about 9% of the total tumor area positive for FAP (Supplemental Fig. 22). PDX2494 was evaluated by PET/CT imaging with [^68^Ga]Ga-DOTA-4AH29 and compared with U87MG xenografts according to an established protocol (Fig. 5; Supplemental Fig. 23) (16). One hour after injection of [^68^Ga]Ga-DOTA-4AH29, PDX2494 yielded decreased tumor uptake (1.34 ± 0.75 %IA/cm^3^) and a decreased tumor-to-muscle ratio (3.29 ± 1.07) compared with U87MG (2.54 ± 0.75 and 8.19 ± 3.46 %IA/cm^3^, respectively). A biodistribution study with [^177^Lu]Lu-DOTA-4AH29 was performed on a small PDX2494 cohort (Supplemental Fig. 24). Lower tumor uptake was confirmed, and mouse dosimetry revealed a 30-fold decrease in tumor MAD (2.0 cGy/MBq) compared with U87MG xenografts (Table 2; Supplemental Table 7).

**TABLE 2.** Mouse Dosimetry of [^177^Lu]Lu-DOTA-4AH29 in PDX2494

Organ	[^177^Lu]Lu-DOTA-4AH29 (cGy/MBq)
Blood	0.21
Kidneys	141.44
Tumor	1.98

MADs were calculated using biodistribution data (Supplemental Fig. 24).

### Pretargeting ^177^Lu-FAP sdAb Therapy Results in Prolonged Survival in a PDAC PDX Model

**FIGURE 6.**
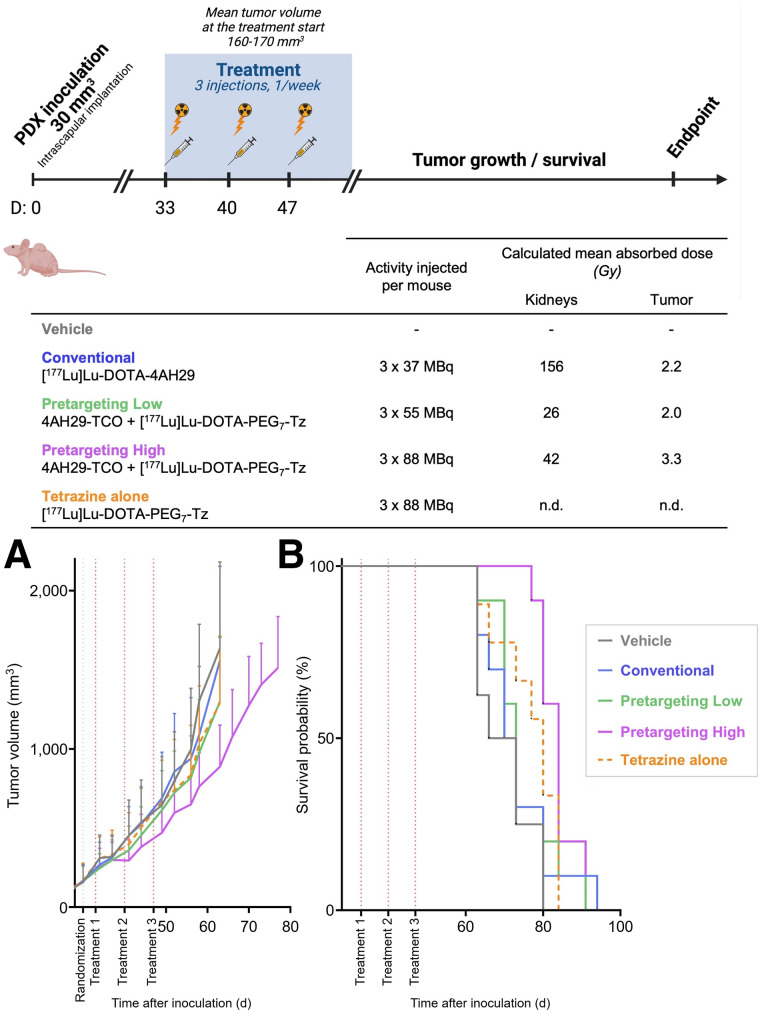
Therapy study in PDX2494-P5 with schematic overview of therapeutic schedule and dosimetry. (A and B) Tumor growth control (A) and Kaplan–Meier plot of survival probability (B) as function of time. Mice were euthanized when tumor volume was >2,000 mm^3^. Survival data reflect progression of primary tumors, because no adverse effects led to anticipated animal euthanasia. n.d. = not determined; Tz = tetrazine.

Mice were treated over a 3-wk period, with treatment once per week and injected activity ranging from 37 to 88 MBq (Fig. 6). The conventional and the pretargeting low-injected-activity cohorts demonstrated similar tumor MAD, whereas kidney MAD was drastically lower for pretargeting. Because of this lower kidney MAD, a pretargeting high-injected-activity cohort was added, allowing the delivery of higher tumor MAD. A control group with the tetrazine radioligand injected alone was used. Overall, the pretargeting high cohort demonstrated improved tumor growth control after treatment, in contrast to the other cohorts (Supplemental Fig. 25). Median survival was significantly longer in the pretargeting high cohort than in the vehicle (*P* = 0.0005), conventional (*P* = 0.0051), and pretargeting low (*P* = 0.0096) cohorts (Table 3). Despite no significant difference between the median survival of the pretargeting high and tetrazine-alone cohorts, we highlight that the tetrazine-alone cohort did not result in significantly prolonged survival of animals compared with the vehicle cohort, confirming the superiority and specificity of the pretargeting high group. Moreover, the tetrazine-alone cohort aligned with controls in terms of tumor growth delay. A transient animal weight decrease on therapy initiation was observed for the pretargeting high cohort, with a less pronounced effect in other treated cohorts and no observed effect in both control groups (Supplemental Fig. 26). Hematologic parameters were monitored. Despite high injected activity, only a transient decrease in white and red blood cells was reported in the pretargeting high cohort, with values remaining in the range of standard healthy mice (Supplemental Fig. 27). Mouse kidneys were collected on animal euthanasia (≤60 d after therapy start), weighted, and stained for histopathologic evaluation. No significant decrease in kidney weight was observed in any cohort (Supplemental Fig. 28). Histopathologic evaluation highlighted a background level of mononuclear cell infiltrate and basophilic tubuli, with an increase of this background pathology in the conventional cohort (Supplemental Table 8). Moreover, the conventional cohort demonstrated mesangial expansion in 7 of 10 mice. Such an observation suggests treatment-related kidney toxicity. At this time point, the pretargeting high cohort presented histopathologic evaluation similar to that of the vehicle control.

**TABLE 3.** Median Survival After Inoculation and Statistical Analysis of PDX2494 on Defined Therapeutic Regimen

Parameter	Vehicle	Conventional	Pretargeting low	Pretargeting high	Tetrazine alone
Median survival (d)	69.5	71.5	73	84	80
*P* value					
Vehicle		NS (0.5552)	NS (0.2909)	0.0005*	NS (0.0742)
Conventional			ND	0.0051^†^	ND
Pretargeting low				0.0096^†^	ND
Pretargeting high					NS (0.0641)
Tetrazine alone					

* *P* ≤ 0.001.

† *P* ≤ 0.01.

NS = nonsignificant; ND = not determined.

*P* values were determined using log-rank Mantel–Cox test.

## DISCUSSION

sdAbs possess unique pharmacologic properties for theranostic applications in oncology (23). Their small size is associated with renal excretion (24). If advantageous for diagnostic purposes, this excretion route is a limitation for TRT. Recent findings by Dekempeneer et al. (16) revealed dose-dependent histopathologic changes in kidneys of naïve mice on administration of [^225^Ac]Ac-DOTA-4AH29, with adverse changes starting at a cumulated administered activity of 30 kBq. Consequently, injected activity for sdAb TRT is limited by the kidney TI, and approaches that reduce kidney retention are being explored. It is even more critical in the context of FAP TRT, a hot topic in nuclear medicine, in which quinoline-based FAPIs showed limited responses because of their fast tumor clearance (25*,*^26^). Therefore, sdAbs appear as an alternative to small-molecule inhibitors to prolong tumor retention.

In this study, we optimized a click-chemistry pretargeting approach with sdAbs to mitigate renal accumulation. The choice of the TCO–tetrazine reactive couple was based on its previous successful application to reduce antibody-related hematotoxicity after TRT (11) and its current clinical evaluation for application in PDAC PET imaging with an antibody conjugate (NCT05737615). Key pretargeting parameters were optimized. First, we reported that TCO functionalization had no major impact on the 4AH29 pharmacokinetic profile. Then, the reduction of kidney retention with pretargeting was confirmed in healthy animals. Evaluation in U87MG (FAP^+^) subcutaneous xenografts validated the 8-h lag time and 200-µg injected mass of 4AH29-TCO as ideal parameters for maximized kidney TI. In comparison to previously reported pretargeting approaches, the injected mass of the sdAb-TCO conjugate falls in the same range as that of antibodies (8*,*^27^). As expected, the optimal lag time is shorter than for antibodies because of the faster clearance of sdAbs. Reduced tumor-cumulated activity was reported with pretargeting compared with [^177^Lu]Lu-DOTA-4AH29. This observation is likely due to a partial receptor-mediated sdAb internalization that leaves TCO unreactive. Nevertheless, we reported a maximum kidney TI of 2.0 ± 0.4 with pretargeting and a 5-fold increase compared with the conventional approach. Such TIs were equalized or surpassed only by particular radiohalogenated sdAbs that presented faster renal clearance, most likely because of the formation of fast-clearing radiocatabolites (16*,*^28^*,*^29^). Comparison between studies is rendered difficult because of differences in the sdAb used, animal models, target expression, or injected mass of sdAb for in vivo evaluation. To our knowledge, the pretargeting kidney TI is the highest for an sdAb–radiometal conjugate reported in the literature.

In this study, the choice of animal models was based on 2 factors: the cohort size and the relevance of the radiobiologic behavior of the targeted cells. To optimize pretargeting parameters, large cohorts of animals were needed and, consequently, a model was required that could be easily generated without consideration of the radiobiologic relevance of the targeted cells. U87MG subcutaneous xenografts presented endogenous FAP expression. However, we noticed dynamic expression of FAP among the cohorts generated throughout the course of this study. Even if tumor-to-kidney ratios were not affected, we reported overall lower tumor uptake when we reproduced our main pretargeting optimization study (Supplemental Fig. 17). Similar observations were recently reported by our group (16) and others (30*,*^31^). Side-by-side comparisons presented in this study were performed on animals originating from the same cohort. For the therapy study, the relevance of the radiobiologic behavior of the targeted cells was prioritized. In patient samples, FAP is expressed on CAFs, a heterogeneous cell population of the tumor microenvironment with a characteristic radiobiologic behavior. Unlike cancer cells, CAFs can survive severe stress, including irradiation at a high absorbed dose, and are recognized as radioresistant cells (32). On exposure to clinically relevant high absorbed doses of external beam radiation therapy, CAFs demonstrated persistent DNA damage, inhibition of their proliferation, and induction of a senescent phenotype (33–35). PDXs were screened for FAP expression because of their superiority in recapitulating the spatial structure and heterogeneity of patients’ cancer. A PDAC PDX, PDX2494, showed appreciable FAP expression on murine CAF infiltrates. Compared with U87MG xenografts, PDX2494 demonstrated lower tumor uptake, which correlates with its lower percentage of FAP-positive tumor area (9% vs. >40%). Because our priority was to target CAFs, a therapy study was initiated in this model, with a dosing regimen adapted from past experiences. Because of PDX2494’s low FAP expression, high activity was administered with corresponding low tumor MAD and penalizing kidney MAD for the conventional and pretargeting high cohorts. Nevertheless, this study validated the therapeutic potential of pretargeting FAP sdAb TRT, with tumor growth delay and prolonged survival for the cohort treated with the highest injected activity. These results are even more appreciable in light of the low tumor MAD delivered. We hypothesize that a greater differential in survival benefit between treated and control cohorts would be observed with a model with higher FAP expression. A longer tumor growth delay was reported with [^131^I]I-GMIB-4AH29 in U87MG xenografts (16) or with [^177^Lu]Lu-FAP2286 in a sarcoma PDX with high FAP expression on tumor cells (36), further highlighting how the targeted cell plays a crucial role in tracer accumulation, tumor MAD, and ultimately antitumor effect. A PDX model with a slower growth curve might also result in a more pronounced therapeutic effect. Overall, our study emphasizes the need for animal models with higher stromal density, more representative of the clinical situation. No morphologic kidney changes were reported with pretargeting, whereas mesangial expansion was reported for the conventional approach. Assessment at later time points might be needed, because kidney toxicity is usually a late effect. On the basis of the extremely high dissociation constant reported for 4AH29 (16) and the improvement of TI with pretargeting, we expect our approach to compete with current FAPIs or peptides.

## CONCLUSION

This study validates the potential of pretargeting to mitigate kidney retention with sdAb–radiometal conjugates and alleviate the renal toxicity associated with sdAb TRT. Key pretargeting parameters optimized in this study should be transferable to other sdAbs. Despite the reported tumor growth delay and prolonged survival with pretargeting in our PDAC PDX model, our study highlights the need for more representative animal models to evaluate stroma–TRT approaches. The clinical future of pretargeting FAP sdAb TRT will strongly depend on the selection of patients with sufficient FAP expression to benefit from the therapeutic effect of the approach.

## DISCLOSURE

We acknowledge the Fondation ARC and the Région Occitanie for funding fellowships. The group and Réseau d’Histologie Expérimentale de Montpellier are supported by SIRIC Montpellier Cancer Grant INCa-DGOS-INSERM-ITMO Cancer 18004 and by Ligue contre le cancer. Yana Dekempeneer, Laurent Navarro, Francis Santens, Nina Dumauthioz, and Matthias D’Huyvetter are employees and Tony Lahoutte is consultant of Precirix NV; all hold ownership interest (including patents) in sdAb radiotherapeutics. Sophie Poty, Matthias D’Huyvetter, Tony Lahoutte, and Jean-Pierre Pouget have filed patent EP22305656 based on the reported results. No other potential conflict of interest relevant to this article was reported.

## Notes

KEY POINTS **QUESTION:** Can pretargeting be applied to sdAbs to reduce kidney retention? **PERTINENT FINDINGS:** Key pretargeting parameters were optimized using FAP sdAb and resulted in a significant decrease in kidney-cumulated activity compared with the conventional approach. Pretargeting resulted in prolonged survival in a PDAC PDX model without signs of renal toxicity compared with the conventional cohort. **IMPLICATIONS FOR PATIENT CARE:** Pretargeting opens the realm of clinical sdAb TRT by increasing kidney TI.
